# Cell‐free biomimetic osteochondral scaffold for the treatment of knee articular surface lesions: Clinical outcomes differ based on patient and lesion characteristics

**DOI:** 10.1002/ksa.12402

**Published:** 2024-08-05

**Authors:** Luca De Marziani, Angelo Boffa, Luca Andriolo, Alessandro Di Martino, Iacopo Romandini, Luca Solaro, Stefano Zaffagnini, Giuseppe Filardo

**Affiliations:** ^1^ Clinica Ortopedica e Traumatologica 2, IRCCS Istituto Ortopedico Rizzoli Bologna Italy; ^2^ Faculty of Biomedical Sciences Università Della Svizzera Italiana Lugano Switzerland

**Keywords:** cartilage lesions, gender, knee, lesion site, scaffold

## Abstract

**Purpose:**

A cell‐free biomimetic osteochondral scaffold was developed to treat cartilage knee lesions, with positive clinical results documented in small case series. However, clear evidence on patient and lesion characteristics that might affect the outcome is still lacking. The aim of this study is to analyse a large cohort of patients treated with this scaffold to investigate factors that could influence the clinical outcome.

**Methods:**

Two hundred and three patients (mean age 30.7 ± 10.9 years) treated with this scaffold were prospectively evaluated at baseline, 6‐, 12‐ and 24‐month follow‐up. The clinical outcome was analysed using the International Knee Documentation Committee (IKDC) score, and the activity level was assessed with the Tegner score. The influence of patient and lesion characteristics on clinical outcomes was analysed.

**Results:**

Mild and severe adverse reactions were found in 39.0% and 1.5% of patients, respectively. The failure rate was 2.0%, increasing to 12.3% when including also clinical failures. The IKDC subjective score increased from 43.3 ± 15.9 to 61.0 ± 16.2 at 6 months, 68.3 ± 18.5 at 12 months and 73.8 ± 18.3 at 24 months (*p* < 0.0005). The Tegner improved from 2.5 ± 1.7 to 4.2 ± 1.7 at 24 months (*p* < 0.0005), without reaching the pre‐injury level (6.0 ± 2.2) (*p* < 0.0005). The IKDC objective score changed from 68.5% normal and nearly normal knees before the treatment to 90.1% at 24 months. At 24 months, age showed a correlation with the IKDC subjective score (*ρ* = −0.247; *p* < 0.0005), women had a lower score (*p* < 0.0005), as well as patients with patellar lesions (*p* = 0.002). Previous surgery correlated with lower results (*p* = 0.003), while better results were found in osteochondritis dissecans (OCD) compared to degenerative lesions (*p* = 0.001).

**Conclusion:**

This cell‐free biomimetic scaffold is a safe and effective treatment for cartilage knee lesions, offering positive clinical results at 2 years with a low failure rate. Better outcomes were observed in younger patients, in lesions of the femoral condyles and in OCD, while joints affected by patellar lesions, patients who underwent previous knee surgery, and women may expect lower results.

**Level of Evidence:**

Level III, cohort study.

**Abbreviations**
ACI: autologous chondrocyte implantation
ACL: anterior cruciate ligament
BMI: body mass index
GLM: general linear model
ICRS: International Cartilage Regeneration and Joint Preservation Society
IKDC: International Knee Documentation Committee
MACT: matrix‐assisted autologous chondrocytes transplantation
OCA: osteochondral allograft transplantation
OCD: osteochondritis dissecans

## INTRODUCTION

Cartilage lesions are a debilitating pathology with a high prevalence, present in 63% of patients undergoing knee arthroscopy [14, 15, 47]. These lesions are frequently localized in the knee due to the high loads and complex functions performed by this joint. If not adequately treated, knee chondral defects can further degenerate, leading to the development of early osteoarthritis [30, 48, 50]. Along with cartilage damage, the subchondral bone is frequently involved, and there is an increasing awareness of its role in pathological processes and the need to address it as well [18, 44]. To this end, different treatments have been developed over the years to manage both chondral and osteochondral layers using autologous or allogeneic osteochondral units or by adopting cell‐based regenerative procedures [6, 49]. Nevertheless, all these approaches present limitations such as donor site morbidity, limited availability, or high costs [29, 31, 52].

Cell‐free scaffolds were developed to overcome these limitations, with the aim of providing a biomimetic and biodegradable three‐dimensional structure able to promote the regeneration of both the chondral layer and the underlying subchondral bone [5, 39, 40]. They demonstrated the capability to serve as stimuli for the differentiation of local bone marrow stromal cells by inducing an ‘in situ’ tissue regeneration without the need for any cell augmentation [41]. Among these scaffolds, an osteochondral scaffold composed of type I collagen and hydroxyapatite has been introduced in clinical practice to address cartilage lesions. This biomaterial showed positive results in terms of safety and effectiveness in small series of patients up to long‐term follow‐ups [18, 51]. The appropriateness of using this type of scaffold was also highlighted in a recent International Cartilage Regeneration and Joint Preservation Society (ICRS) Consensus [25], although the real potential and indications for this scaffold remain supported by limited literature evidence and still rather by expert opinions. Clear data for profiling patient and lesion characteristics that might affect the outcome and guide the surgeon in treatment choice are still missing. In this light, the analysis of a broad population of patients affected by articular surface lesions treated with a cell‐free osteochondral scaffold would be clinically relevant, allowing the identification of factors influencing the results provided by this surgical approach.

The aim of this study is to prospectively analyse a large cohort of patients treated with a cell‐free biomimetic scaffold to document its safety and efficacy for up to 2 years and to investigate patient and defect characteristics that could influence the clinical outcome when addressing articular surface knee lesions. The hypothesis is that this treatment presents overall good outcomes and that patient and joint‐related factors can influence the clinical results offered by this cell‐free biomimetic scaffold.

## MATERIALS AND METHODS

### Patient selection

This study was approved by the hospital Ethics Committee of the IRCCS Istituto Ortopedico Rizzoli, Bologna, Italy (Prot. no. 0009122) in accordance with the Declaration of Helsinki. Informed consent was obtained from all patients. Inclusion criteria were patients with clinical symptoms such as knee pain or swelling and with International Cartilage Repair Society (ICRS) Grades 3–4 chondral and osteochondral lesions or osteochondritis dissecans (OCD) involving the femoral condyles, trochlea, or patella that underwent scaffold implantation between January 2007 and January 2022. The procedures were performed in a specialized orthopaedic clinic and by a surgeon team expert in the treatment of cartilage lesions. Exclusion criteria were untreated malalignment or ligament instability or other general medical conditions at the time of surgery including infections, neoplastic, metabolic and inflammatory pathologies. Patients with axial malalignment or ligament instability underwent a realignment procedure or ligament reconstruction during the same surgical session of the scaffold implantation.

A total of 219 consecutive patients were enrolled and treated in accordance with the inclusion/exclusion criteria. Among them, 203 patients were prospectively evaluated at baseline, 6, 12 and 24 months of follow‐up (16 were lost to follow‐up). Eighty‐seven patients were surgically treated for the first time, while 116 patients had undergone previous surgeries, including 51 meniscectomies, 30 anterior cruciate ligament (ACL) reconstructions, 25 microfracture techniques, 20 surgical treatments for knee fractures, 17 knee arthroscopies, 14 loose body removals, 14 procedures of chondral lesion shaving, 14 previous cartilage treatments, 3 patellar realignments and 2 knee osteotomies. In 96 patients, other combined procedures were performed during the same operation: 26 knee osteotomies, 20 patellar realignments, 11 meniscectomies, 9 ACL reconstructions, 7 microfracture techniques, 7 loose body removals, and 14 other procedures (lateral releases, patellar tendon suturing, meniscal allograft, meniscal implants, patellar lateral facet removal, lateral tibial plateau elevation and hardware removal). All demographic and clinical characteristics are reported in detail in Table 1.

**Table 1:** Included patients' characteristics.

Sex, M/W	155/48
Age, y	30.7 ± 10.9
BMI, kg/m^2^	24.3 ± 2.9
Symptoms duration, m	45.5 ± 54.4
Previous knee surgery, yes/no	116/87
Concomitant knee surgery, yes/no	96/107
Kellgren–Lawrence grade	Grade 0: 46
	Grade 1: 119
	Grade 2: 29
	Grade 3: 9
	Grade 4: 0
Lesion size, cm^2^	3.2 ± 2.1
Lesion location	MFC: 90
	LFC: 49
	Patella: 47
	Trochlea: 29
Number of lesions	Single: 192
	Multiple: 11
Aetiology	Traumatic: 40
	Degenerative: 87
	OCD: 76

*Note*: Values are expressed as mean ± standard deviation.

Abbreviations: BMI, body mass index; LFC, lateral femoral condyle; MFC, medial femoral condyle; M, men; m, months; OCD, osteochondritis dissecans; W, women; y, years.

### Scaffold characteristics

This osteochondral biomimetic scaffold (Maioregen, Fin‐ceramica Faenza Spa, Faenza, Ita) is a three‐layer material which mimics osteochondral anatomy [33]. In detail, the cartilage layer is made up of type I equine collagen and has a smooth surface to facilitate the sliding of the joint surface. The middle layer is similar to the tide mark of the cartilage and is made of a combination of type I collagen (60%) and hydroxyapatite (40%), while the lower layer is made of type I collagen (30%) and hydroxyapatite (70%), which reproduces the subchondral bone layer. The scaffold is available in different formulations, the classic rectangular formulation that can be customized and shaped freehand and, alternatively, three pre‐formed cylindrical sizes (1.2, 1.5 and 1.8 cm in diameter) implantable with dedicated instrumentation [2].

### Surgical procedure and post‐operative protocol

After applying a tourniquet to the proximal part of the thigh, the incision of skin, subcutaneous layers and capsule can be made medially or laterally to the patella, depending on the lesion location. Once the chondral or osteochondral lesion is visualized, the lesion is prepared and the injured cartilage is removed to create an area of specific depth to place the scaffold of the desired size. According to the measure of the lodging, the right size of the scaffold is chosen. Then the scaffold can be inserted with a press‐fit technique. In the end, fibrin glue can be applied, if necessary according to the surgeon's decision case by case, to achieve greater stability [2].

Post‐operative care depended on the position of the treated lesion. For the condyles lesions, knee flexion and isometric and isotonic muscle strengthening exercises were allowed from the first post‐operative days, while a restricted loading was permitted for the first 3 or 4 weeks, after which the patient could progressively load. For patellofemoral lesions, knee flexion exercises were allowed after a week, while isometric and isotonic muscle strengthening exercises, in addition to partial load, were allowed for the first 2 weeks and afterwards the patient could progressively load.

### Patient evaluation

All patients were prospectively clinically evaluated at baseline and at 6, 12, and 24 months after treatment. The clinical outcome was analysed using the International Knee Documentation Committee (IKDC) score, both subjective and objective. The sports activity level was assessed at baseline (pre‐injury and pre‐operative values) and at the last follow‐up using the Tegner score.

Adverse events and failures were also recorded by interviewing the patients at all follow‐up visits. Mild adverse events were defined as the presence of fever or significant pain or swelling of the treated knee for over 7 days as reported by patients, and severe adverse events were defined as any event that resulted in death, was life‐threatening, or required hospitalization or interventions to prevent permanent impairment or damage. The procedures were considered to have failed if the patient needed a reoperation for the same defect during the follow‐up time including a revision of the implant or knee arthroplasty. For these patients, the worst clinical evaluation between baseline and the last available follow‐up was considered for the following assessments. In accordance with previous literature, we considered a clinical failure based on the general knee condition score reported by the patient, which did not remain steadily improved (>10 points) from baseline to final follow‐up during two consecutive 1‐year intervals [20].

### Statistical methods

All continuous data were expressed in terms of the mean and the standard deviation of the mean, the categorical data were expressed as frequency and percentages. The Shapiro–Wilk test was performed to test the normality of continuous variables. The repeated measures general linear model (GLM) with Sidak test for multiple comparisons was performed to assess the differences at different follow‐up times of the quantitative scores. The Friedman non‐parametric test followed by the Wilcoxon pairwise comparisons with Bonferroni correction was performed to assess the differences at different follow‐up times of the ordinal scores. One‐way ANOVA with the Scheffé post hoc pairwise analysis was performed to assess differences among groups when the Levene test for homogeneity of variances was not significant (*p* < 0.05); otherwise, the Mann–Whitney *U* test (two groups) or the Kruskal–Wallis test with the non‐parametric post hoc pairwise Dunnet test was used. The Spearman rank correlation was used to assess correlations between quantitative scores and continuous data. The Kendall–Tau Correlation was used to assess correlations between quantitative or ordinal scores and ordinal data. The Fisher chi‐square exact test was performed to assess the relationship between dichotomous variables. The Pearson chi‐square evaluated using exact test was performed to investigate relationships between categorical variables. For all tests, *p* < 0.05 was considered significant.

## RESULTS

Mild adverse reactions such as post‐operative fever or knee swelling for more than 7 days were found in 78 patients (38.5%), as reported in Table 2. In addition, three patients (1.5%) presented post‐operative joint stiffness requiring an arthroscopic arthrolysis, while four patients (2.0%) were considered failed. Among treatment failures, three patients underwent osteochondral autograft transplantation at 2, 3 and 12 months of follow‐up, respectively, while one patient underwent partial knee replacement at 24 months of follow‐up. Moreover, other 21 patients were identified as clinical failures, for a total of 12.3% of patients considering both surgical and clinical failures.

**Table 2:** Patients' adverse events.

	Patients	Percentage (%)
No adverse events	122	60
Fever	34	16.5
Swelling	49	24
Stiffness	3	1.5

*Note*: The total % is not 100 since some patients had more than one adverse event, es. five patients had both fever and swelling (2.5%).

**Figure 1:**
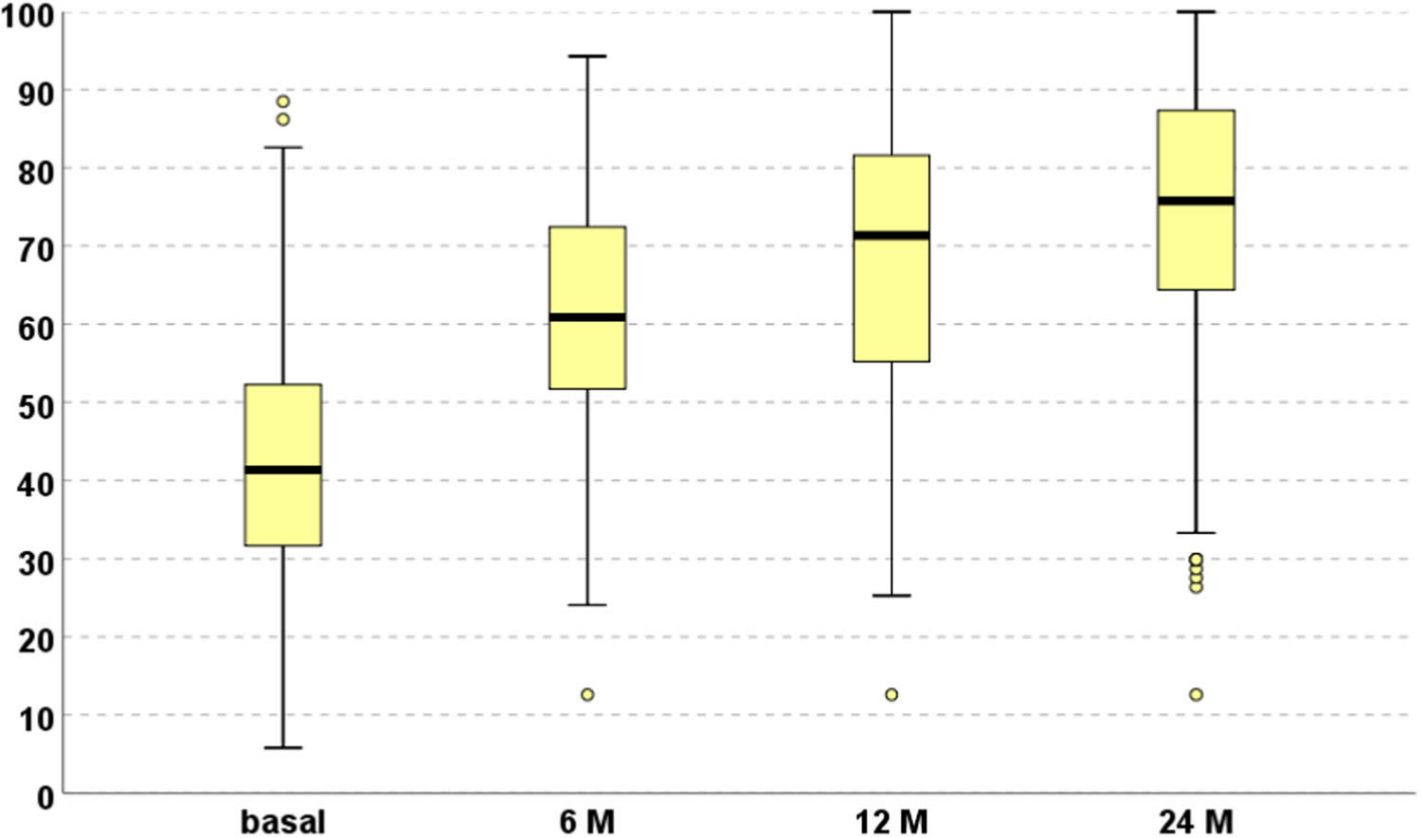
IKDC subjective score (0–100) at baseline level and at 6, 12 and 24 months of follow‐up. IKDC, International Knee Documentation Committee.

An overall significant improvement in all scores was found after the treatment. The IKDC subjective score (Figure 1) significantly increased from the baseline level of 43.3 ± 15.9 to 61.0 ± 16.2 at 6 months, 68.3 ± 18.5 at 12 months and 73.8 ± 18.3 at 24 months (all *p* < 0.0005 compared to baseline). A statistically significant improvement was observed from 6 to 12 months (*p* < 0.0005) and from 12 to 24 months (*p* < 0.0005).

The IKDC objective score (Table 3) changed from 68.5% normal and nearly normal knees before the treatment to 86.2% at 6 months, 90.1% at 12 months and 90.1% at 24 months of follow‐up (all *p* < 0.0005 compared to baseline). A statistically significant improvement was observed from 6 to 12 months (*p* < 0.0005) and from 12 to 24 months (*p* = 0.024).

**Table 3:** IKDC objective score and Tegner score.

IKDC objective score	Baseline	6 months	12 months	24 months
	A: 79	A: 94	A: 120	A: 143
	B: 60	B: 81	B: 63	B: 40
	C: 42	C: 17	C: 13	C: 15
	D: 22	D: 11	D: 7	D: 5
Tegner score	2.5 ± 1.7	3.0 ± 1.0	3.8 ± 1.6	4.2 ± 1.7

*Note*: Objective International Knee Documentation Committee (IKDC) is reported as number of patients, while the Tegner score is reported as mean ± standard deviation.

**Figure 2:**
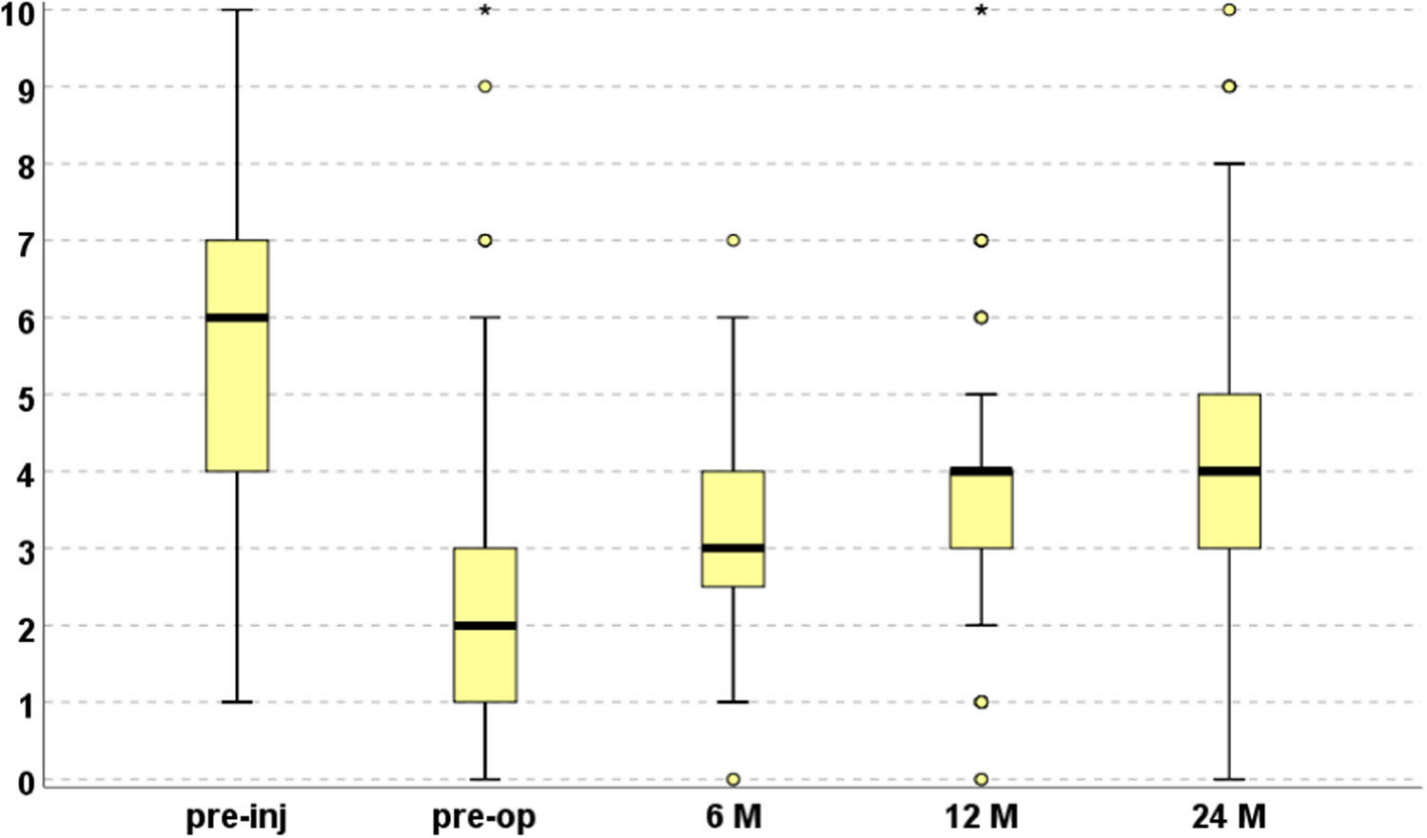
Activity level evaluated with the Tegner score at pre‐injury and pre‐operative level, and at 6, 12 and 24 months of follow‐up.

The Tegner score (Figure 2) significantly improved from the pre‐operative to 6, 12 and 24 months of follow‐up (all *p* < 0.0005 compared to baseline). A statistically significant improvement was observed from 6 to 12 months (*p* < 0.0005) and from 12 to 24 months (*p* < 0.0005). However, the Tegner score did not reach the same pre‐injury level (6.0 ± 2.2) at any follow‐ups (all *p* < 0.0005 compared to the pre‐injury level). Only 75 patients (37%) reached at the last follow‐up the same pre‐injury activity sport level. Further analysis was performed to evaluate the parameters that might influence the clinical outcome. Age showed a negative correlation with the IKDC subjective score at all evaluations, with higher IKDC subjective scores found in younger patients: baseline (*ρ* = −0.158; *p* = 0.024), 6 months (*ρ* = −0.290; *p* < 0.0005), 12 months (*ρ* = −0.241; *p* = 0.001) and 24 months (*ρ* = −0.247; *p* < 0.0005). Sex influenced the clinical outcome with worse results in women compared to men. In detail, women had a lower IKDC subjective score compared to men at baseline (39.1 ± 17.0 vs. 44.6 ± 15.4; *p* = 0.036) and at 6 months (55.1 ± 16.4 vs. 62.8 ± 15.8; *p* = 0.005), 12 months (59.9 ± 19.0 vs. 70.9 ± 17.5; *p* < 0.0005) and 24 months (65.4 ± 18.7 vs. 76.4 ± 17.3; *p* < 0.0005) of follow‐up. Moreover, women had a lower Tegner score compared to men pre‐injury (4.5 ± 2.3 vs. 6.5 ± 2.0; *p* < 0.0005), pre‐operatively (1.9 ± 1.7 vs. 2.6 ± 1.6; *p* = 0.012) and at 6 months (2.5 ± 1.0 vs. 3.2 ± 1.0; *p* < 0.0005), 12 months (3.0 ± 1.6 vs. 4.0 ± 1.5; *p* < 0.0005) and 24 months (3.4 ± 1.3 vs. 4.4 ± 1.7; *p* < 0.0005) of follow‐up. However, while the absolute scores in men and women at baseline and follow‐up showed significant differences, no differences were documented for both IKDC and Tegner scores when comparing the amount of improvement obtained from baseline at the follow‐ups.

The defect site influenced the clinical results, with lower clinical outcomes in patients affected by patellar lesions. In fact, while no differences were detected in the baseline scores, patellar lesions showed a lower IKDC subjective score compared to lateral femoral condyle lesions at 12 months (*p* < 0.0005) and at 24 months (*p* = 0.002). Moreover, patellar lesions showed a lower Tegner score compared to lateral femoral condyle lesions at 12 months (*p* = 0.026) and at 24 months (*p* = 0.001) and compared to medial femoral condyle lesions at 24 months (*p* = 0.012). Moreover, lateral femoral condyle lesions had a higher IKDC subjective score compared to medial femoral condyle lesions (*p* = 0.035) at 12 months, but not at the last follow‐up. The subjective scores at different follow‐ups for each lesion site are reported in detail in Table 4.

**Table 4:** Scores at the different follow‐up times for each lesion site.

Lesion site	Baseline		6 Months		12 Months		24 Months	
	IKDC	Tegner	IKDC	Tegner	IKDC	Tegner	IKDC	Tegner
MFC: (90)	45.8 ± 15.8	2.9 ± 1.7	62.2 ± 16.0	3.2 ± 1.1	67.5 ± 18.1	3.7 ± 1.5	72.6 ± 18.3	4.2 ± 1.7
LFC: (49)	44.5 ± 17.9	2.5 ± 2.1	65.1 ± 15.0	3.0 ± 1.1	76.2 ± 16.2	4.3 ± 1.8	80.8 ± 16.6	4.7 ± 1.8
Patella: (47)	40.0 ± 16.0	1.8 ± 1.0	56.7 ± 17.2	2.8 ± 1.1	60.8 ± 18.2	3.3 ± 1.3	67.1 ± 17.1	3.4 ± 1.1
Trochlea: (29)	40.8 ± 12.9	2.1 ± 1.4	58.1 ± 15.3	2.9 ± 1.0	68.5 ± 19.4	3.5 ± 1.6	74.9 ± 20.0	4.4 ± 1.9

*Note*: Values are expressed as mean ± standard deviation.

Abbreviations: LFC, lateral femoral condyle; MFC, medial femoral condyle; IKDC, International Knee Documentation Committee.

**Figure 3:**
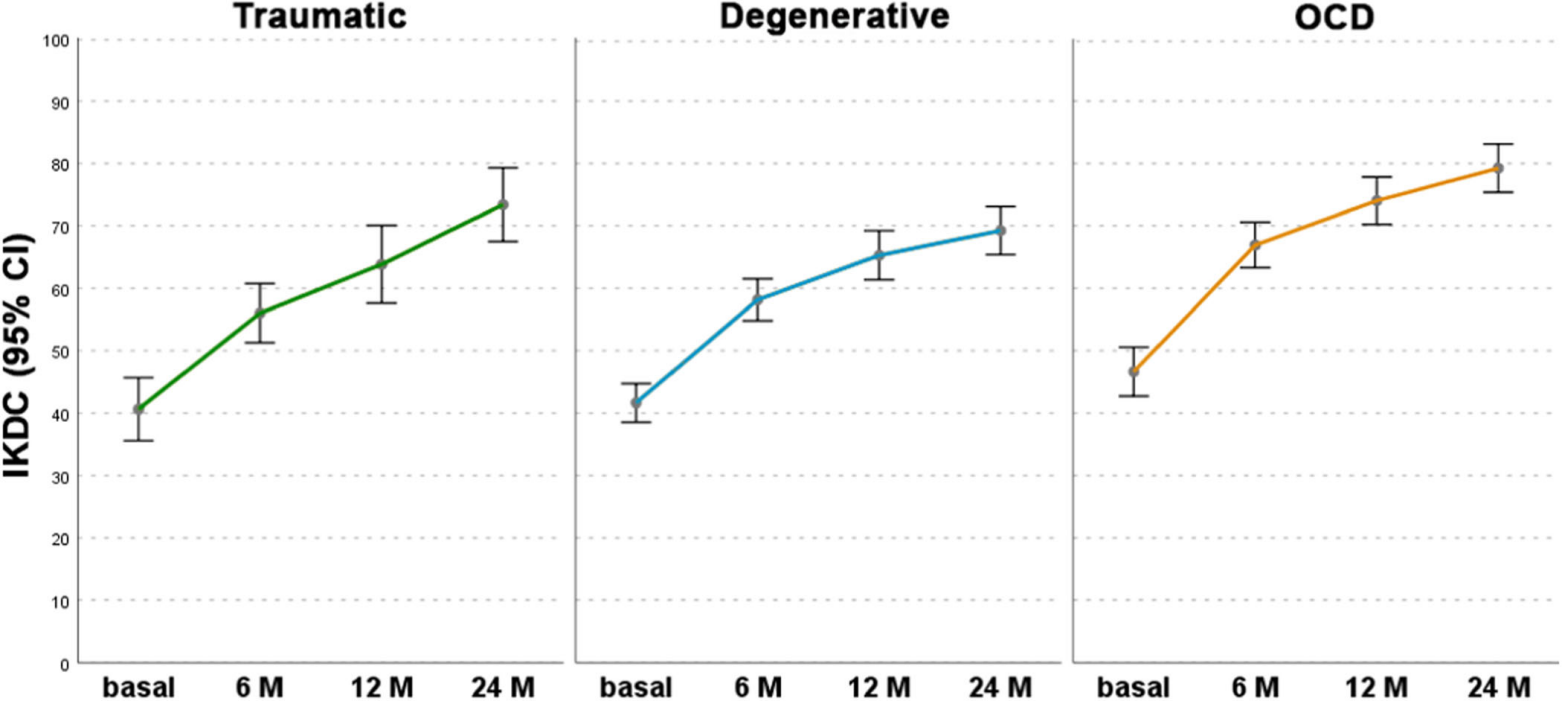
IKDC subjective score in degenerative (green), traumatic (orange) and OCD (blue) lesions. Presented as mean values and 95% CI of the mean. CI, confidence interval; IKDC, International Knee Documentation Committee; OCD, osteochondritis dissecans.

The aetiology of the lesions influenced the clinical outcomes, with overall better results in patients affected by OCD lesions compared to traumatic and degenerative lesions (Figure 3). In detail, OCD lesions showed a higher IKDC subjective score compared to degenerative lesions at 6 months (66.9 ± 15.9 vs. 58.1 ± 15.7; *p* = 0.002), 12 months (74.0 ± 16.6 vs. 65.3 ± 18.6; *p* = 0.005) and 24 months (79.3 ± 17.0 vs. 69.3 ± 18.3; *p* = 0.001), and a higher IKDC subjective score compared to traumatic lesions at 6 months (66.9 ± 15.9 vs. 56.0 ± 14.8; *p* = 0.001) and 12 months (74.0 ± 16.6 vs. 63.8 ± 19.5; *p* = 0.019). Patients with post‐operative adverse events showed a lower improvement from baseline to 12 months in the IKDC subjective score compared to patients without post‐operative adverse events (+21.0 ± 18.8 vs. +27.6 ± 20.4; *p* = 0.01), while no differences were found at 24 months. Patients who underwent previous knee surgery showed a lower IKDC subjective score at 24 months of follow‐up compared with patients without previous surgery (71.1 ± 19.3 vs. 77.4 ± 16.0; *p* = 0.003). BMI, lesion area, Kellgren–Lawrence grade and the presence of combined surgery did not influence the clinical outcome or the post‐operative adverse events in this series.

## DISCUSSION

The main finding of this study is that this cell‐free biomimetic scaffold is a safe and effective treatment for knee articular surface lesions, offering positive results in terms of subjective and objective clinical improvement at 2 years with a low failure rate. However, not all patients benefited the same and several factors have been identified with prognostic value. Overall better outcomes were observed in younger patients, lesions of the femoral condyles, and OCD, while women patients who underwent previous knee surgery and joints affected by patellar lesions presented lower results.

The current study prospectively analysed over 200 patients treated with this scaffold, confirming the promising clinical results observed in the previous small preliminary studies, providing a progressively increasing clinical improvement from baseline up to 2 years. Moreover, a very low failure rate was observed, with only 2% of the patients requiring surgery at the short‐term follow‐up. This was lower compared to the failure rate reported for other cartilage procedures at the same follow‐up. In fact, a systematic review analysed different cartilage surgical procedures, including microfracture, autologous chondrocyte implantation, osteochondral autograft transplantation, and osteochondral allograft, reporting a surgical failure rate at 2 years of 14.7%, 29.7%, 8.8% and 12.2%, respectively [26]. On the other hand, a high rate of post‐operative adverse events (39%) was observed after the implantation of this scaffold, although only 1.5% of patients experienced severe adverse events, and these had no impact on the clinical outcomes at 2‐year follow‐up. In fact, patients with post‐operative adverse events showed a lower clinical improvement from baseline to 12 months, but no further differences were found at the last follow‐up in terms of both clinical outcome and activity level.

The patient activity level significantly improved compared to the pre‐operative level. Nevertheless, the post‐operative activity level did not reach the same level observed before the onset of symptoms with only 37% of patients returning to the pre‐injury level. This finding confirms previous studies on this scaffold [33, 34], underlining the need for careful communication with patients on the return‐to‐sport expectations. Patients are likely to experience satisfactory improvements in symptoms and function, but they should have realistic expectations regarding their activity levels 2 years after the procedure. Return‐to‐sport after cartilage procedures is often incomplete, as reported by a recent meta‐analysis on over 2500 high‐level athletes that documented a 75% return to sport at mid‐term follow‐up [38]. The lower results observed in this series may be due to the fact that the population was not composed solely of athletes. Furthermore, the presence of different cartilage lesion etiologies including degenerative lesions sometimes in joints already suffering from osteoarthritis processes could influence the results. A previous study on 27 patients affected by OCD and treated with the same scaffold demonstrated a return to the same pre‐injury activity level at a longer follow‐up of 5 years [46]. Moreover, a study [45] on 20 patients (most of them affected by OCD) reported that patients returned to the same pre‐injury activity level at an average follow‐up of 47 months after autologous chondrocyte implantation (ACI). In this light, it would be crucial to evaluate the patients of this series at longer follow‐ups to analyse if there is an improvement in the return to sport over time, also in relation to the type of patients and lesions.

This study showed that patients affected by OCD had overall better clinical outcomes and activity levels compared to patients affected by traumatic and degenerative cartilage lesions, confirming the positive results suggested by previous studies [4, 17]. Moreover, a multicenter randomized trial on 100 patients showed better results at 2 years when treating OCD lesions with this scaffold compared to those obtained with bone marrow stimulation [35]. A better response to cartilage treatment in patients with OCD has also been observed for other cartilage procedures. A systematic review by Chahla et al. [10] on 644 knees treated with osteochondral allograft transplantation (OCA) and evaluated at an average follow‐up of 58 months reported that OCD showed more favourable results compared to other etiologies. Similarly, Andriolo et al. documented better clinical results after arthroscopic matrix‐assisted autologous chondrocyte transplantation (MACT) in patients with OCD lesions compared to patients affected by degenerative cartilage lesions [1]. The better clinical results observed in OCD found in the current study and in other studies on other cartilage procedures can be attributed to a different healing potential of these lesions and also to a better state of the surrounding cartilage compared to degenerative lesions [46]. In fact, in a chronic degenerative process, adjacent areas are likely to be involved, with cytokines produced by the chondrocytes around the implant that may cause dedifferentiation or apoptosis and, overall, a less favourable environment for tissue regeneration [21, 42].

Gender was identified as another important factor significantly impacting the clinical outcomes in this case series. Women showed overall lower clinical scores and also lower levels of physical activity than men. Similar results have been reported by the German Cartilage Registry on 4968 patients treated with different cartilage treatments, with lower clinical scores, greater dissatisfaction following treatment, and a higher rate of revision surgery at 2 years in women [19]. This registry analysis also highlighted that women often present a different cartilage lesion pattern, with more frequent conditions, such as degenerative lesions and patellar cartilage lesions, which could complicate their treatment [8, 14, 30]. The lesion site, in fact, represents another key aspect for cartilage surgery, with patellar cartilage lesions representing the most challenging site for cartilage regeneration [12, 16, 27]. Bartha et al. [3] analysed the results of 831 mosaicplasty procedures performed in different joints and reported the worst results in the patello‐femoral cartilage lesions, with 79% of good‐to‐excellent results compared to 92% in condylar lesions. Similar results were also found for ACI, with Kreuz et al. [37] reporting that female patients with femoral lesions had significantly better results than patients with patello‐femoral lesions at mid‐term follow‐up. In a further analysis on patello‐femoral lesions, Filardo et al. [23] demonstrated the particularly lower outcome specific to patellar lesions versus trochlear lesions treated with MACT, with women being more commonly affected by this challenging lesion type [22]. The current study confirmed that both women and patellar lesions treated with this scaffold present poorer clinical outcomes. The main reason could be the combination of high forces during flexion and multidirectional stresses of the patella in the patellofemoral joint, depending on the patellar and trochlear shape, muscle forces, ligaments knee stability, and alignment [36]. However, while reaching lower final scores, it is important to also underline that patellar lesions still presented an overall significant and clinically relevant improvement exceeding what is currently considered a minimal clinically significant difference after treatment of this type of lesions [11].

Age is another prognostic factor after treatment with this cell‐free biomimetic scaffold, with poorer outcomes observed in older patients. The negative effect of age on cartilage treatment was already documented for other cartilage procedures [13, 36, 43]. A recent analysis by Byrne et al. [9] on a database of 6391 patients treated with OCA showed that older patients presented a higher risk of failure. A study by Andriolo et al. [1] on 113 patients affected by cartilage lesions of the femoral condyles and trochlea treated with MACT reported worse outcomes at 15 years of follow‐up in older patients. Comparative studies between microfractures and ACI or mosaicplasty also reported better clinical results in patients aged under 30 years, regardless of the type of treatment performed [28, 32]. An explanation for these findings could be related to the lower regenerative potential and synthetic function of chondrocytes in older patients, on the one hand, but, on the other hand, also to the overall lower scores reached when applying the evaluation scores in older cohorts in the healthy population, reflecting a natural decline of function and activity level over time [7, 24, 43].

This study investigates prospectively a large patient cohort treated by the same specialized team with the same osteochondral scaffold and provides important new insights, but also presents some limitations. The lack of a control group represents the major limitation of this study, impairing the possibility to understand the real benefits offered by this cell‐free biomimetic scaffold compared to other available cartilage procedures. However, a previous comparative clinical study already established the superiority of this scaffold over microfractures, which is still considered the reference technique to compare cartilage procedures [35]. Furthermore, the lack of imaging evaluation avoided the possibility of assessing the characteristics of the repair tissue and evaluating radiological features that could influence the clinical results. Nevertheless, this aspect has been explored in previous studies [18, 33, 53], which never showed a correlation between clinical and radiological data. In this perspective, the patients' clinical condition after treatment remains the primary outcome. Finally, it is important to underline the heterogeneity of the clinical characteristics of the patients evaluated, particularly in terms of aetiology, lesion size, previous and concomitant procedures, as well as post‐op protocols and surgeons. On the other hand, this heterogeneity was essential in reflecting the real‐world scenario and allowing to identify patient prognostic factors that could influence the results. Despite the aforementioned limitations, the current study provided robust data on the clinical outcome obtained with this biomimetic scaffold, although further long‐term studies are needed to confirm its potential in terms of clinical benefit and survival rate over time in the different patient and lesion categories.

## CONCLUSIONS

This study demonstrated that this cell‐free biomimetic scaffold is a safe and effective treatment for knee articular surface lesions, offering positive results in terms of subjective and objective clinical improvement at 2 years with a low failure rate. Overall, better outcomes were observed in younger patients, with lesions of the femoral condyles and OCD, while joints affected by patellar lesions, patients who underwent previous knee surgery, and women may expect lower results.

## AUTHOR CONTRIBUTIONS

Conceptualization: Giuseppe Filardo. Methodology: Luca Andriolo, Angelo Boffa and Alessandro Di Martino. Formal analysis and investigation: Luca De Marziani, Iacopo Romandini and Luca Solaro. Writing—original draft preparation: Luca De Marziani and Angelo Boffa. Writing—review and editing: Luca Andriolo, Angelo Boffa, Alessandro Di Martino, Giuseppe Filardo and Luca Solaro. Supervision: Giuseppe Filardo and Stefano Zaffagnini.

## FUNDING INFORMATION

The authors declare that no funds, grants, or other support were received for this study.

## CONFLICT OF INTEREST STATEMENT

Stefano Zaffagnini is a consultant surgeon for Smith and Nephew and DePuy Synthes. He has received institutional support from Fidia Farmaceutici, Cartiheal, IGEA Clinical Biophysics, Biomet and Kensey Nash, grant support from I+, and royalties from Springer outside the submitted work. The other authors declare no conflict of interest.

## ETHICS STATEMENT

This study was performed in line with the principles of the Declaration of Helsinki and approved by the Local Ethics Committee of the IRCCS Istituto Ortopedico Rizzoli (approval protocol no. 0009122). All the enrolled patients signed the informed consent.

## Data Availability

The Ethics Committee did not authorize sharing the raw patients' data. The calculated average values, which protect patients' privacy, are detailed in the manuscript.
